# Ultrasound imaging of the perineal body: a useful clinical tool

**DOI:** 10.1007/s00192-019-04166-7

**Published:** 2019-12-11

**Authors:** Victoria Asfour, Giuseppe Alessandro Digesu, Ruwan Fernando, Vik Khullar

**Affiliations:** St Mary’s Hospital, Imperial College Healthcare NHS Trust, Paddington, London, W2 1NY UK

**Keywords:** Perineal body, Prolapse, Theory of prolapse, Perineum

## Abstract

**Introduction and hypothesis:**

The perineal body is a fibromuscular pyramidal structure located between the vagina and the anus. It has been difficult to image because of its small size and anatomical location. This study used 2D transperineal ultrasound to measure the perineal body and assess whether there is an association with prolapse.

**Methods:**

An observational, cross-sectional study was carried out in a tertiary level Urogynaecology department and included prolapse patients and healthy nulliparous volunteers (control group). This was a clinical assessment, including POP-Q and trans-perineal 2D ultrasound measurement of the perineal body height, length, perimeter, and area. Parametric tests were used, as the data were normally distributed. Results are reported as mean and 95% confidence interval (±95% CI).

**Results:**

A total of 101 participants were recruited of which 22 were nulliparous healthy volunteers. Mean perineal body measurements in controls were height 22.5 ± 3.3 mm, length 17.4 ± 2.7 mm, perimeter 7.5 ± 0.9 mm, and area 2.8 ± 0.38 cm^2^. Perineal body measurements in 79 prolapse patients: height 16.9 ± 1.7 mm, length 16.0 ± 1.4 mm, perimeter 6.5 ± 0.5 mm and area 2.1 ± 0.5 cm^2^. A small perineal body was strongly associated with posterior compartment prolapse (paired* t* test, *p* < 0.0001) and wider POP-Q GH (paired* t* test, *p* = 0.0003). Surprisingly, Pelvic Organ Prolapse Quantification Perineal Body (POP-Q PB) of the two groups was not significantly different. A perineal body mid-sagittal area of less than 2.4 cm^2^ has been shown to be associated strongly with posterior compartment prolapse.

**Conclusions:**

It is possible to measure the perineal body on 2D ultrasound. This technique facilitates the objective diagnosis of perineal deficiency. POP-Q PB does not predict the length or area of the perineal body.

## Introduction

The perineal body is a fibromuscular pyramidal structure that is located between the vagina and the anus. The perineal body is a confluence of multiple muscle attachments. Laterally, it extends into the transverse perineal muscles that insert into the ischium. Anteriorly, the posterior vaginal wall and fourchette are located. Anterolaterally, it fuses with the bulbospongiosus muscle. Posteriorly is the anal canal. The external anal sphincter and the perineal body fuse in the midline. At the superior apex boundary, it fuses with the puborectalis portion of the pubovisceralis muscle (also known as the levator ani muscle).

Historically, in the 1880s, Emmet and in the 1910s, Kelly described the posterior repair and perineorrhaphy that are still performed today. They emphasised that performing a perineorrhaphy as an important component of posterior prolapse repair [1, 2].

Several theories of prolapse have been proposed, including DeLancey’s theory, Petros’ theory and Tansatit’s theory [3].

DeLancey in the 1990s suggested that the uterus might be suspended by a fascial hammock connecting the cervix from the pubis to the sacrum [4]. He describes three levels of support for the female genital tract, where the perineum is at level 3. It is suggested that failure at this level might be due to a disruption from the endopelvic fascia and bulbocavernosus muscles [5].

Petros’ integral theory, published in 2008, endorses DeLancey’s hammock theory and suggests that the main support of the vaginal walls is the collagen binding the tissues together [6]. He proposes that the pelvic floor musculature further supports the fascial hammock. Therefore, disruption to the collagenous structures places undue strain on the rest of the system, leading to pelvic organ prolapse.

Tansatit’s theory, published in 2013, suggests a support system based on levator ani muscle injury, the arcus tendinous fascia pelvis and ischio-anal fossa adipose tissue [7]. The appearance of levator muscle avulsion injury has been associated with prolapse [8]. The ultrasound appearance of levator ani avulsion injury has been clearly demonstrated to occur at childbirth; however, this finding often resolves several months or years later [9].

Perineal body mobility was evaluated in pregnant women in the third trimester and 3–6 months after birth [10]. In that study, perineal body location was identified on the midsagittal plane of 3D volume scans, where the images included a full view of all the pelvic floor structures. Perineal body mobility was measured and compared before and after childbirth, by assessing the location of the perineal body to the horizontal plane of the pubic symphysis. They found that the mobility of the perineal body increased after childbirth [10].

In 2016, the perineal body of nulliparous cadavers was visualised using a three-dimensional endovaginal ultrasound ((3D-EVUS) BK high frequency ultrasound scanner, with 3D Viewer offline analysis [11]. In the EVUS scan, the perineal body is visualised as an ovoid structure.

Magnetic resonance imaging (MRI) has been extensively used to assess the posterior compartment. However, even though a number of studies have examined the posterior compartment in prolapse, not many have assessed the perineal body [12]. An MRI study examined the perineal body in 11 asymptomatic women [13]. The perineal body was identified and reconstructed from multiple sections using offline 3D computer modelling. They found that on MRI, the perineal body appears to be a pyramid; however, it was not possible to delineate it with clarity for detailed assessment of dimensions, at any level (superficial, mid-portion, deep) [13].

Traditionally, the perineal body has been thought of as being the central tendon of the perineum. However, it has been shown that this is a misnomer, because the perineal body is a confluence of muscles, but histologically, it is not a tendon [14].

The role of the perineal body and its association with vaginal prolapse has not been formally evaluated. Studies have attempted to visualise the perineal body using MRI, CT and ultrasound. The perineal body is difficult to visualise owing to its small size and anatomical location.

The aim of this study was to measure the perineal body using 2D ultrasound and assess whether the dimensions may vary in the presence of prolapse.

## Materials and methods

Ethical approval was granted by the Riverside Ethics committee IRAS 17/LO/1398. Health Research Authority approval was granted. Prolapse patients were recruited from the urogynaecology clinic. Healthy volunteers were recruited with the aid of leaflets in waiting areas in the hospital. All the patients had a clinical assessment, including a POP-Q and an ultrasound scan. A curved 7 MHz 2D probe (AB27D) was used with the GE Voluson™ E8 scanner.

All patients on the waiting list for prolapse surgery were included. Exclusion criteria included poor English, lack of capacity to consent and lack of consent to the study.

Patients were asked to void prior to the scan. In patients with severe prolapse, the prolapse organs were re-positioned in the vagina after the POP-Q assessment. Vaginal pessaries were removed for the assessment.

**Fig. 1:**
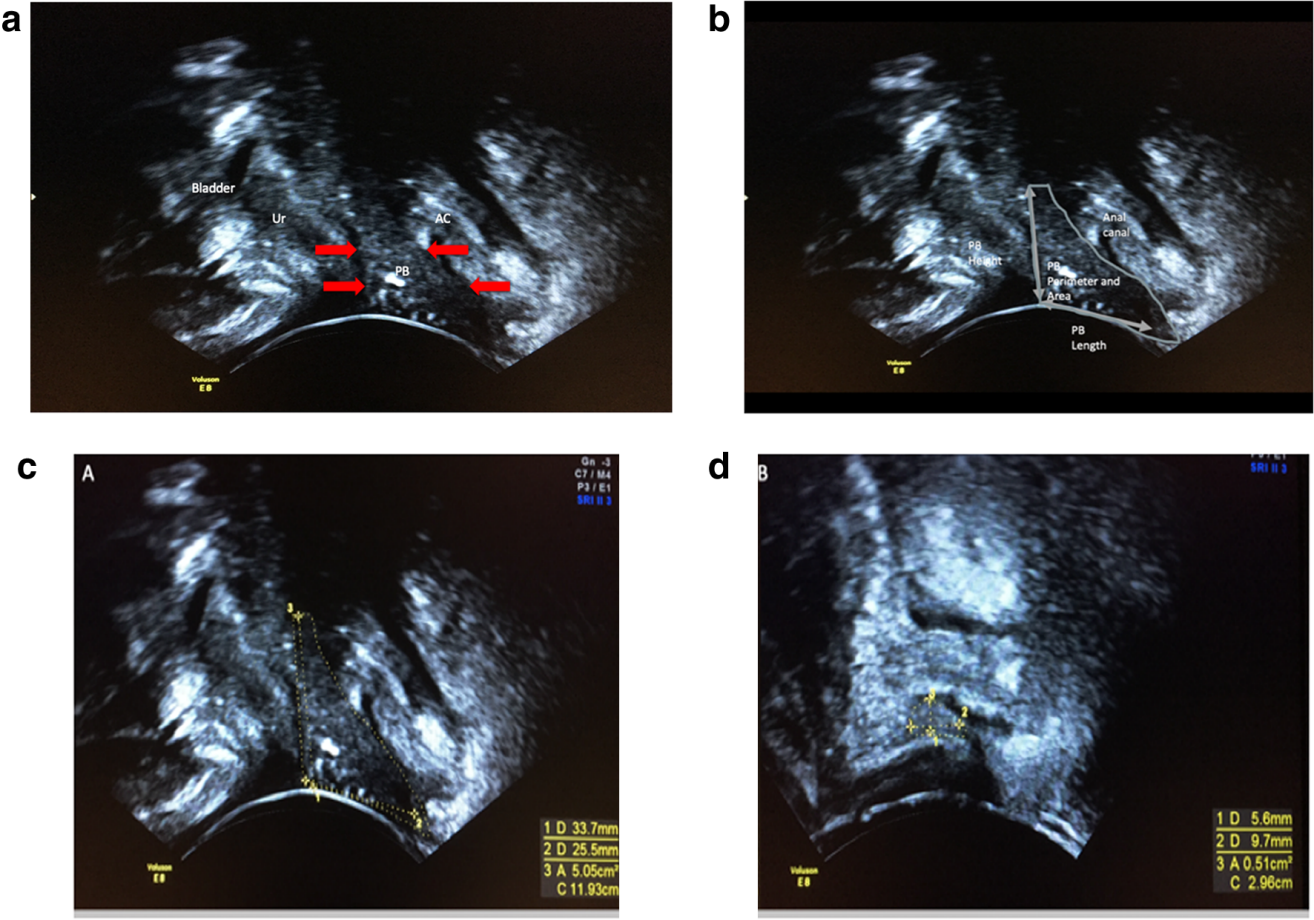
**a**) Image of a normal perineal body from a healthy volunteer. The perineal body is shown with red arrows. **b**) Perineal body(PB) measurements are outlined (Length, Height and Perimeter). **c**) Perineal body measurements in a healthy volunteer. **d**) Perineal body scan measurements in a prolapse patient

The women were scanned in a supine position with legs abducted. The probe was placed on the perineum. The image was inverted, so that the cranial part of the patient was at the top of the screen, and the bladder moved downwards on Valsalva. The scanning angle was opened to 107°. The pelvic floor was visualised in the mid-sagittal plane. Small adjustments were made to minimise the echo cast by the pubis anteriorly and to image the urethra, the perineal body and anal canal in the largest and longest dimensions. The perineal body image was then maximally magnified and optimised by adjusting the Gain, so that each image consisted mainly of the perineal body. The perineal body was measured in length, height, perimeter and area (Fig. 1). The height of the perineal body is the cranio-caudal dimension, which appears like the vertical side of a triangle. The length of the perineal body is the antero-posterior dimension, which is the base of the triangle. The perimeter was a manual trace of the pyramidal edge of the perineal body in the mid-sagittal plane. The area was calculated by the scanner at the end of the perimeter trace. The first set of measurements was taken during bedside scanning. A second set of measurements was taken at an interval time blinded to the previous values for inter-observer and intra-observer variation.

For statistical analysis, SPSS version 25 was used. Data are presented as mean ± standard deviation (SD) and 95% confidence interval (CI). Test for normality was done using the Shapiro–Wilk test. Student’s* t* test was used for univariate and one-way ANOVA with Bonferroni post hoc analysis was performed for multivariate data to assess the difference in size of the perineal body and POP-Q. A *p* value of <0.05 was deemed statistically significant. Validation testing was performed using Bland–Altman analysis.

## Results

One hundred and one patients were recruited for this study. Seventy-nine were consecutive patients on the waiting list for prolapse repair.

The control group consisted of 22 healthy nulliparous non-pregnant volunteers. All our healthy volunteers had also attended the recurrent miscarriage clinic. The healthy volunteers had a mean age of 33.8 years and body mass index (BMI) 24. The prolapse group had an average age of 58.5 years and BMI 28.9. In prolapse patients, 57 had a cystocoele, 49 had a rectocoele and 39 had apical prolapse.

All the measurements displayed a normal distribution. Shapiro–Wilk test for the perineal body ultrasound measurements was 0.8 for length, 0.7 for height, 0.1 for perimeter and 0.4 for area.

Comparisons of POP-Q and perineal body ultrasound measurements in healthy volunteers and prolapse patients are found in Tables 1 and 2.

**Table 1:** Pelvic Organ Prolapse Quantification (*POP-Q*) comparison of symptomatic prolapse patients with asymptomatic, nulliparous, non-pregnant controls. The measurements were compared using a paired *t* test

POP-Q	Mean	SD	SE mean	*p* value
PB POP	2.98	0.98	0.13	0.7
PB N	3.06	0.64	0.21	
GH POP	3.39	1.27	0.27	0.003
GH N	2.31	0.70	0.15	
TVL POP	9.64	1.59	0.33	0.6
TVL N	9.82	1.33	0.28	
D POP	−7.05	2.46	0.52	0.002
D N	−9.18	1.29	0.28	
Aa POP	−1.33	1.52	0.33	<0.0001
Aa N	−3.00	0.00	0.00	
Ba POP	−1.76	1.33	0.29	<0.0001
Ba N	−3.00	0.00	0.00	
C POP	−5.23	0.97	0.21	0.008
C N	−1.14	2.18	0.31	
Ap POP	−1.14	2.17	0.47	0.003
Ap N	−2.86	0.48	0.10	
Bp POP	−1.00	2.62	0.57	0.02
Bp N	−2.95	0.22	0.48	

*PB* perineal body,* POP* pelvic organ prolapse,* GH* genital hiatus,* TVL* total vaginal length,* SD* standard deviation,* SE* standard error. N (healthy volunteers), POP (prolapse patients)

**Table 2:** Perineal body (*PB*) ultrasound measurements in healthy volunteers and prolapse patients. The measurements were compared using a Student’s* t* test

	Normal			Prolapse			
	Mean	SD	CI	Mean	SD	CI	*p* values
PB length (mm)	17.4	6.4	20.1–14.7	16.0	7.6	17.4–14.6	<0.0001
PB height (mm)	22.5	7.8	25.8–19.2	16.9	8.7	18.6–15.2	<0.0001
PB perimeter (mm)	7.5	2.2	8.4–6.6	6.5	2.9	7–6	<0.0001
PB area (cm^2^)	2.8	1.1	3.3–2.3	2.1	1.6	2.3–1.9	<0.0001

*SD* standard deviation, *CI* confidence interval

The perineal body was defined as “small” when it was less than 2.4 cm^2^, which is below the lower limit of the 95% confidence interval. Forty-nine out of 79 prolapse patients (62%) had a small perineal body of less than 2.4 cm^2^.

The POP-Q PB measurements of the control and prolapse groups were not found to be significantly different. The genital hiatus was found to be significantly increased in patients with prolapse. POP-Q scores were significantly associated with a small perineal body (less than 2.4 cm^2^), with POP-Q GH (*p* = 0.0003) and posterior vaginal measurements being most strongly associated with a small perineal body (*p* < 0.0001).

The measurements were validated for inter-observer and intra-observer variability (Tables 3, 4).

**Table 3:** Control patients: perineal body (*PB*) validation

Parameter	*n*	Mean	Mean difference	Standard deviation	*t *test* p* values	95% CI lower value	95% CI upper value	Linear regression* p* values
PB length inter-observer variation	19	17.1	−0.2	1.8	0.6	−1.1	0.6	0.9
PB length intra-observer variation	19	17.1	−0.1	1.4	0.8	−0.7	0.6	0.7
PB height inter-observer variation	19	25.8	−0.7	2.1	0.1	−1.8	0.3	0.9
PB height intra-observer variation	19	26.8	−0.9	2.7	0.1	−2.2	0.3	0.4
PB perimeter inter-observer variation	19	8.2	−0.1	0.4	0.3	−0.3	0.1	0.9
PB perimeter intra-observer variation	19	8.3	−0.1	0.6	0.3	−0.4	0.1	0.2
PB area inter-observer variation	19	2.9	0.02	0.3	0.8	−0.1	0.2	0.4
PB area intra-observer variation	19	2.9	−0.01	0.3	0.9	−0.2	0.1	0.2

*CI* confidence interval

**Table 4:** Prolapse patients: validation with the smallest perineal bodies (*PB*)

Parameter	*N*	Mean	Mean difference	Standard deviation	*t* test* p* values	95% CI lower values	95% CI upper values	Linear regression* p* values
PB length inter	19	10.2	0.03	1.8	0.9	−0.8	0.9	0.6
PB length intra	19	9.8	0.4	1.2	0.2	−0.2	1.1	0.5
PB height inter	19	11.9	−0.4	1.6	0.3	−1.2	0.4	0.9
PB height intra	19	12.1	−0.3	0.9	0.3	−0.7	0.2	0.7
PB length inter	19	4.3	−0.3	0.4	0.8	−0.3	0.2	0.9
PB length intra	19	4.3	−0.6	1.4	0.8	−0.7	0.6	0.7
PB perimeter inter	19	4.3	−0.03	0.5	0.8	−0.3	0.2	0.1
PB perimeter intra	19	4.3	0.006	0.4	0.9	−0.2	0.2	0.3
PB area inter	19	0.9	−0.02	0.2	0.6	−0.2	0.1	0.2
PB area intra	19	0.9	−0.03	0.1	0.2	−0.1	0.01	0.02

*CI* confidence interval

## Discussion

To the best of our knowledge, this is the first description of accurately measured dimensions of the perineal body on 2D ultrasound. This is a simple, non-invasive method of imaging the perineal body that can be performed in the clinic. The values of the perineal body dimensions were obtained during live scanning. This trans-perineal ultrasound scan can be performed in a clinical outpatient setting with a 7-MHz curved linear probe, such as those found in most modern Obstetrics and Gynaecology departments. This method of scanning the perineal body gives the clearest images observed so far in the literature. Previous studies had shown poor repeatability [10, 11]. This may be because, in those studies, the perineal body was measured on images that acquired the entire pelvic floor. When measuring small structures by ultrasound, such as the nuchal translucency or a gestational sac, it is important for the image to be optimised for these measurements. We acquired maximally zoomed-in images that were optimised specifically for the perineal body. Optimised images enable the accurate and repeatable measurement of the perineal body.

In prolapse, the perineal body area is less than 2.4 cm^2^, as defined by the lower value of the 95% confidence interval. A small perineal body (less than 2.4 cm^2^) is associated with prolapse.

The POP-Q PB does not correlate with the perineal body length on ultrasound, as demonstrated by these data and those of previous studies [15]. The POP-Q PB only considers the length between the fourchette and the anus, but does not consider the thickness of the substance of this structure, which in cases of prolapse may be just skin. The ultrasound was aimed at assessing this area in more detail. The perineal body is known to be a confluence of the pelvic floor muscles. This study has shown that in prolapse, this confluence is damaged. It is found to be smaller in prolapse patients.

Other studies correlating POP-Q PB and POP-Q GH with the presence of prolapse have also shown similarly that increasing stages of prolapse are associated with POP-Q GH, but not with POP-Q PB [16]. POPQ PB + GH have been shown to improve the prediction of levator injuries in women with incontinence and prolapse [17].

The POP-Q PB may not correlate with perineal body ultrasound measurements owing to the presence of perineocoeles. Perineocoeles have been demonstrated in cadaveric anatomy studies [18]. In these cases, the perineal muscles weaken and sublux, causing the bowel to prolapse into the perineum [18]. A perineocoele may co-exist with a rectocoele. Clinically, this is not immediately evident because the POP-Q PB distance does not discriminate between these entities.

These data suggest that the perineal body might be an important support structure for the anorectal complex. A small perineal body (less than 2.4 cm^2^) is strongly associated with posterior compartment prolapse. These findings are in support of Emmet’s and Kelly’s thinking, in which they emphasised the importance of perineorrhaphy, i.e., perineal body reconstruction in vaginal prolapse repair [1, 2].

These findings also supplement the theories for prolapse discussed above. Failure of the collagenous infrastructure puts the musculature of the pelvic floor under strain. The perineal body is the confluence of many pelvic floor muscles. It is possible that when put under strain from other structural changes, such as levator ani injury or collagen fascial compromise, the confluence of muscles is slowly pulled apart, leaving the anorectum unsupported. This possibility is supported by cadaver dissection work showing subluxation of the muscles that would normally attach on the perineal body [18].

Alternatively, it may be that primary damage of the perineal body, which may be a destabilising factor in itself, putting the fascial collagenous structures under undue strain, causing them to fail over time. For example, as with levator ani injury, the perineal body may also be damaged during childbirth [19]. It may also be possible for both of these hypotheses to be true in different women. In advanced prolapse, procidentia, all the structural supports are likely to have failed, resulting in complete eversion of the vagina.

Beyond childbirth-related mechanical disruption, the pathophysiology of prolapse is also known to include multiple collagen gene defects [20], hormone-induced collagen alterations [21], neurological factors [22], changes in the extracellular matrix [23], age [24, 25], obesity [26], constipation [27], chronic pelvic floor stress [28], previous operations (such as hysterectomy) [29], and a wider transverse inlet of the bony pelvis [30].

The strength of this study is the novel ultrasound method for the assessment of the perineal body in the posterior compartment. This technique contributes towards a comprehensive clinical assessment of the pelvic floor.

Future research could further evaluate the role of the perineal body, using the technique described here, in understanding the pathophysiology of prolapse.

## Conclusion

It is possible to obtain clear images of the posterior compartment of the pelvic floor by using 2D trans-perineal ultrasound. The perineal body can be measured on 2D ultrasound in its length, height, perimeter and area. A perineal body area of less than 2.4 cm^2^ has been shown to be strongly associated with posterior compartment prolapse. These data support the historic approach to prolapse, where the perineal body was an important component for the repair of the pelvic floor support system.
